# Cohort profile: trajectory of knee health in runners with and without heightened osteoarthritis risk (TRAIL) in Australia—prospective cohort study

**DOI:** 10.1136/bmjopen-2025-101625

**Published:** 2025-09-28

**Authors:** Danilo De Oliveira Silva, Benjamin F Mentiplay, Michael Girdwood, Melissa J Haberfield, Andrea M Bruder, Adam G Culvenor, Thomas J West, Joshua P Hill, David L Carey, Richard T R Johnston, Kay M Crossley

**Affiliations:** 1 La Trobe Sports and Exercise Medicine Research Centre, La Trobe University - Bundoora Campus, Melbourne, Victoria, Australia; 2 Australian International Olympic Committee (IOC) Research Centre, Melbourne, Victoria, Australia; 3 Physiotherapy Discipline, La Trobe University, School of Allied Health, Human Services and Sport, Melbourne, Victoria, Australia

**Keywords:** Knee, Magnetic Resonance Imaging, Gait Analysis, Observational Study, Orthopaedic sports trauma, SPORTS MEDICINE

## Abstract

**Abstract:**

**Purpose:**

The TRAjectory of knee heaLth in runners (TRAIL) study is a prospective cohort study investigating the long-term knee health trajectories of runners with and without a heightened osteoarthritis risk. This study aims to describe the recruitment results and baseline characteristics of the TRAIL cohort.

**Participants:**

Runners aged 18–50 years and running ≥3 times and ≥10 km per week on average in the past 6 months were eligible. Participants were recruited via running podcasts, running clubs and social media between July 2020 and August 2023. Data were collected at study enrolment and at a face-to-face baseline testing session, which occurred a median of 33 weeks (IQR 18 to 86 weeks) after enrolment. Follow-up data collection is ongoing.

**Findings to date:**

Out of 462 runners who completed an online registration form, 268 runners enrolled, of which 135 had a history of knee surgery (46% females) and 133 were non-surgical controls (50% females). 60% of the surgery group had undergone anterior cruciate ligament reconstruction, 33% meniscus and/or cartilage surgery, and 7% other knee surgery. 54 participants previously enrolled were unable to continue in the study before attending baseline data collection. Of the 214 runners who remained in the study and attended baseline data collection, 108 had a history of knee surgery (49% females) and 106 did not have a history of knee surgery (51% females).

**Future plans:**

Participants will be followed for 10 years through ongoing patient-reported outcomes and continuous monitoring of training loads using wearable devices. At baseline, 4- and 10-year follow-up, knee MRI and knee-health patient-reported outcomes will be collected to evaluate structural and symptomatic knee osteoarthritis progression. Data will inform guidelines for safe running practices and rehabilitation post-knee surgery.

**STRENGTHS AND LIMITATIONS OF THIS STUDY**

- This large cohort study recruited an even split of female and male runners across the surgical and control groups.
- Comprehensive baseline data collection will allow the exploration of numerous factors related to the onset or progression of symptomatic or structural knee osteoarthritis in young to middle-aged runners.
- Ongoing data collection of every run logged by participants via wearable devices (smartwatch) will enable estimation of the effect of running exposure on primary and secondary outcomes.
- The absence of a non-runner control group limits the external validity of the cohort findings to the general population.

## Introduction

 Running is a popular physical activity worldwide, valued for its accessibility and health benefits.^1^ However, the repetitive mechanical loads associated with running may have deleterious effects on knee health, particularly in individuals with a history of knee surgery who then have a heightened risk of early osteoarthritis.^2^,^5^ Understanding the association between running biomechanics, training loads and knee health is crucial,^6^ especially as participation in recreational running is rapidly growing.^7^

It is unclear if running can protect or damage knee structures.^28^,^11^ Longitudinal findings about the impact of running on changes in knee symptoms and structural changes are limited in those with a heightened osteoarthritis risk (eg, following anterior cruciate ligament reconstruction (ACLR) or meniscectomy).^12^,^14^ After knee surgery, many people opt for a perceived safer form of exercise (ie, running rather than pivoting sports) to avoid further injury and/or surgery.^15^ However, these individuals often exhibit altered lower-limb joint mechanics and structure,^14 16^ further complicating the relationship between running and knee health outcomes.

The TRAjectory of knee heaLth in runners (TRAIL) cohort study addresses these evidence gaps by comprehensively investigating the long-term trajectory of knee health in runners with and without a history of knee surgery. By collecting patient-reported outcomes, knee MRI, daily training load monitoring with wearable technology, three-dimensional running biomechanics and other biopsychosocial variables, TRAIL aims to create a robust dataset to explore the long-term relationship between running exposure and knee health outcomes.

This cohort profile outlines the recruitment results, and baseline characteristics of the TRAIL cohort study, which will provide a framework to understand the relationships between running, knee health and the risk of early osteoarthritis. The data obtained by the TRAIL study will inform the design of evidence-based strategies for running-related injury prevention and management.

## Cohort description

TRAIL is a prospective cohort study^17^ that monitors overall knee and musculoskeletal health outcomes and training load of runners with and without a history of knee surgery in Australia.

### Recruitment procedures and eligibility criteria

Participants were recruited via sponsored advertisements in running podcasts (ie, Inside Running Podcast; For the Kudos Podcast) and non-sponsored advertisements at running clubs in Melbourne, Australia and on social media (ie, Instagram, Facebook, X). Interested participants accessed a registration form available at trail.latrobe.edu.au and were contacted by a study investigator for telephone-based eligibility screening (online supplemental file 1).

To be eligible to participate in the TRAIL study, at the time of recruitment, all participants had to: (1) be aged 18–50 years; (2) be running ≥3 times and ≥10 km per week on average in the past 6 months; and for the surgical group; (3) have a history of knee surgery (anterior or posterior cruciate ligament, meniscal, chondroplasty, collateral ligament or arthroscopy). Exclusion criteria were: (1) currently pregnant; (2) contraindications to MRI; (3) unable to understand spoken or written English; for the surgery group; (4) a history of intra-articular knee fracture, arthroplasty, osteotomy, patellar tenotomy or lateral retinacular release; and for the control group; (5) no history of knee surgery; (6) no history of any other lower-limb surgery (eg, hip/ankle) and (7) no history of musculoskeletal traumatic or overuse injury in the last 6 months. After the screening, if eligible, potential participants had the opportunity to consent to be enrolled in the study.

Noteworthy, these eligibility criteria had to be met at the time of recruitment; if, during the study, the participants’ conditions change (eg, pregnancy, injuries, surgeries), we are collecting the information and monitoring the overall knee and musculoskeletal health outcomes and training load of participants.

### Data collection

A detailed description of the data collection procedures and outcomes collected in the TRAIL cohort is described in the published protocol^17^ and onlinesupplemental files 1^3^. In summary, the following data are being collected at various time points within the study (table 1):

- Sociodemographic factors, surgery details and self-reported running behaviour (onlinesupplemental files 1  2).
- Participant clinical characteristics (online supplemental file 3).
- Patient-reported outcomes (online supplemental file 3).
- Knee MRI.
- Trunk and lower limb biomechanics, performance-based functional measures and strength tests.
- Running load through wearable technology (smartwatch).
- Running-related pain.
- Qualitative interviews.^18^
- Biomarkers.
- Dual-energy X-ray absorptiometry.
- Additional domains related to women’s health will be collected at a later time point with data reported in future papers.

Table 1 describes the time points for each data collected (enrolment, baseline, 4-year follow-up from baseline and 10-year follow-up; along with daily, monthly, 6-monthly or yearly data collected between the four main time points).

**Table 1:** Summary of time points and data being collected by the TRAIL study

		Data collection frequency		Data collection frequency		Data collection frequency	
**TRAIL data**	**Enrolment**	**↔**	**Baseline**	**↔**	**4-year follow-up**	**↔**	**10-year follow-up**
Sociodemographic factors, surgery details and participant clinical characteristics	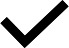		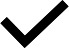		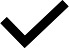		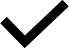
Patient-reported outcomes	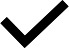		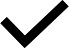	6-monthly	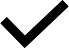	Yearly	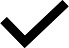
Reference knee MRI			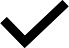		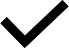		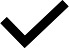
Biomechanics, function and strength			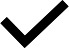				
Running load (smartwatch)	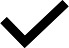	Daily	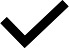	Daily	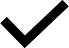	Daily	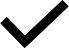
Running-related pain	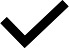	Monthly	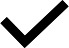	Monthly	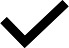		
Qualitative interviews*	Once-off (2021–2022)						
Biomarkers*			Once-off (2024–2025)				
Dual-energy X-ray absorptiometry*			Once-off (2024–2025)				
Women’s health outcomes*			Once-off (2024–2025)				

* Data were collected from a subset of participants.

TRAIL, TRAjectory of knee heaLth in runners.

### Patient and public involvement

Qualitative and quantitative data from participants with a history of ACLR informed the design and development of the research questions of our prospective cohort study, as described in the protocol and our consumer advisory group articles.^6 17^ The views of, and data from, participants following ACLR highlighted the need for this longitudinal prospective study in runners with a history of knee surgery. The TRAIL study also has two ambassadors, Ellie Pashley (Olympian–Tokyo 2020) and Tyler Scarce (competitive community runner), who contributed to media content and study recruitment.

## Findings to date

The findings to date section reports the recruitment outcomes and flow of participants through the study, the participant’s characteristics at the enrolment time point, and general characteristics at the baseline time point, but not the full dataset of all variables (similar to other cohort profile studies).^19^,^22^

### Recruitment data

**Figure 1:**
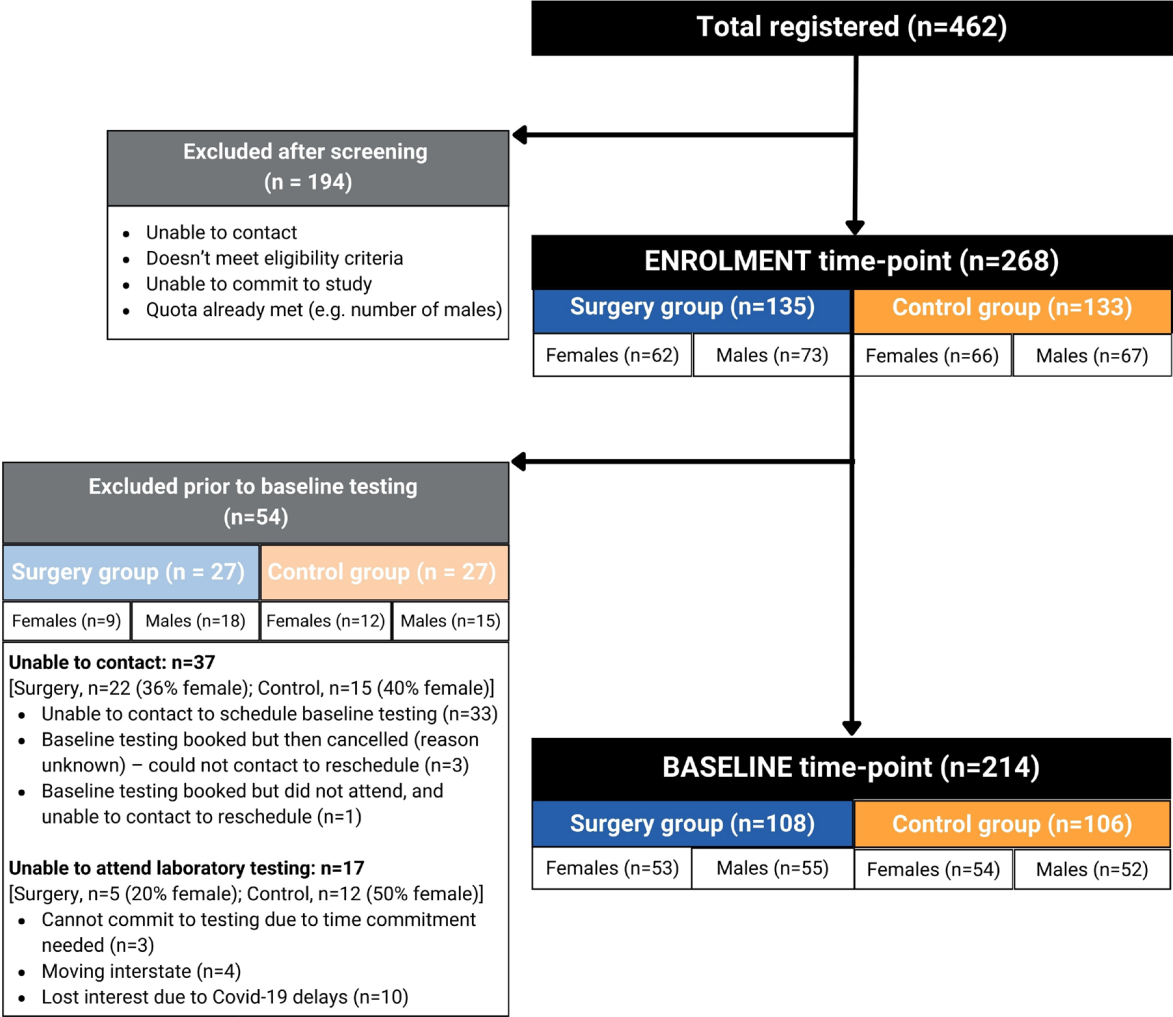
Flow chart describing the TRAjectory of knee heaLth in runners participant’s trajectory through the study.

A total of 462 potential participants completed the TRAIL registration form. After telephone-based screening, 268 participants were enrolled (127 social media, 86 podcasts, 26 TRAIL website, 29 not recorded). They then completed a battery of online questionnaires (sociodemographic and participant characteristics, patient-reported outcomes, running-related pain) and set up their smartwatch at their enrolment time point (figure 1). Due to six COVID-19 pandemic lockdowns in Melbourne, Australia (2020 to 2022), the baseline data collection of the TRAIL study occurred from November 2020 to September 2023. Consequently, 54 previously enrolled participants were unable to attend the baseline data collection and had to cease their participation in the study. Of the 214 who attended the baseline time point, 108 had a history of knee surgery (49% females) and 106 were non-surgical controls (51% females).

We targeted a 1:1 enrolment ratio between the surgery and control groups to support balance between groups; no formal matching was undertaken, and no post-enrolment exclusions were implemented to force balance. Of note, when we reached 85% of the recruitment target established in our protocol,^17^ we reviewed our sex balance and noticed a disparity (59% of participants were males). In an attempt to reach sex parity, aiming for equitable sex representation in research^23^ and to reflect running participation in Australia (where 50% of runners are females),^7^ we focused on the recruitment of females only. Therefore, the last 30 participants included were female runners.

### Enrolment time-point (pre-baseline)

#### Demographics

Participants’ self-reported sociodemographic characteristics can be found in table 2. We present summarised data for all enrolled participants (n=268), for the participants who were excluded prior to the baseline time point (n=54) and for the participants who remained in the study and subsequently completed the baseline data collection (n=214).

**Table 2:** Sociodemographic self-reported characteristics at the enrolment time point

Variable	Enrolled (n=268)		Excluded prior to baseline (n=54)		Completed baseline testing (n=214)	
	Control	Surgery	Control	Surgery	Control	Surgery
	n=133	n=135	n=27	n=27	n=106	n=108
Sex (female/male)	49.6% (66)/50.4% (67)	45.9% (62)/54.1% (73)	46.4% (12)/55.6% (15)	33.3% (9)/66.7% (18)	50.9% (54)/49.1% (52)	49.1% (53)/50.9% (55)
Age, years	32.0±6.7 Median 30.0 (IQR 27.0 to 36.0)	34.2±7.4 Median 33.0 (IQR 28.0 to 40.0)	31.2±5.6 Median 30.0 (IQR 29.0 to 35.5)	35.0±8.0 Median 33.0 (IQR 29.0 to 42.0)	32.1±6.9 Median 30.0 (IQR 27.0 to 36.7)	34.0±7.2 Median 33.0 (IQR 28.0 to 40.0)
Height, m (self-reported)	1.74±0.09 Median 1.73 (IQR 1.67 to 1.80)	1.74±0.09 Median 1.75 (IQR 1.68 to 1.81)	1.75±0.07 Median 1.75 (IQR 1.69 to 1.80)	1.76±0.09 Median 1.77 (IQR 1.68 to 1.79)	1.74±0.10 Median 1.73 (IQR 1.67 to 1.81)	1.74±0.09 Median 1.75 (IQR 1.68 to 1.81)
Body mass, kg (self-reported) (n=266)*	68.3±10.6 Median 68.0 (IQR 60.0 to 75.0)	71.1±11.4 Median 72.0 (IQR 63.0 to 79.0)	68.0±8.0 Median 69.5 (IQR 60.5 to 73.5)	72.4±11.0 Median 72.0 (IQR 65.5 to 78.5)	68.4±11.1 Median 68.0 (IQR 59.0 to 75.0)	70.8±11.6 Median 71.0 (IQR 62.6 to 79.0)
Body mass index, kg/m^2^ (self-reported) (n=266)*	22.4±2.2 Median 22.6 (IQR 20.8 to 23.8)	23.3±2.5 Median 23.4 (IQR 21.7 to 24.7)	22.2±2.1 Median 22.8 (IQR 20.1 to 23.4)	23.4±2.2 Median 23.8 (IQR 21.7 to 24.6)	22.5±2.2 Median 22.6 (IQR 20.8 to 23.9)	23.2±2.6 Median 23.2 (IQR 21.7 to 24.64
Region of birth (n=267)*						
Africa	3.0% (4)	0.7% (1)	3.7% (1)	3.8% (1)	2.8% (3)	0.0% (0)
Americas	2.3% (3)	1.5% (2)	3.7% (1)	0.0% (0)	1.9% (2)	1.9% (2)
Asia	1.5% (2)	1.5% (2)	0.0% (0)	0.0% (0)	1.9% (2)	1.9% (2)
Australia	78.9% (105)	85.1% (114)	70.4% (19)	88.5% (23)	81.1% (86)	84.3% (91)
Europe	9.0% (12)	8.2% (11)	11.1% (3)	3.8% (1)	8.5% (9)	9.3% (10)
Middle East	0.0% (0)	1.5% (2)	0.0% (0)	3.8% (1)	0.0% (0)	0.9% (1)
Oceania (excl. Australia)	5.3% (7)	1.5% (2)	11.1% (3)	0.0% (0)	3.8% (4)	1.9% (2)
Indigenous (n=262)*	0% (0)	0% (0)	0% (0)	0% (0)	0% (0)	0% (0)
Education level (n=266)*						
Doctorate degree	1.5% (2)	4.5% (6)	0.0% (0)	0.0% (0)	1.9% (2)	5.6% (6)
Masters degree	19.7% (26)	18.7% (25)	19.2% (5)	15.4% (4)	19.8% (21)	19.4% (21)
Bachelors degree	62.9% (83)	57.5% (77)	57.7% (15)	53.8% (14)	64.2% (68)	58.3% (63)
Graduate diploma	9.8% (13)	10.4% (14)	15.4% (4)	19.2% (5)	8.5% (9)	8.3% (9)
High school completion	6.1% (8)	8.2% (11)	7.7% (2)	7.7% (2)	5.7% (6)	8.3% (9)
Some high school	0.0% (0)	0.7% (1)	0.0% (0)	3.8% (1)	0.0% (0)	0.0% (0)
Occupation (n=261)*						
Clerical and administrative worker	5.4% (7)	2.3% (3)	3.8% (1)	7.7% (2)	5.8% (6)	1.0% (1)
Community and personal service worker	6.9% (9)	9.9% (13)	7.7% (2)	7.7% (2)	6.7% (7)	10.5% (11)
Labourer	0.8% (1)	0.0% (0)	3.8% (1)	0.0% (0)	0.0% (0)	0.0% (0)
Machinery operators and drivers	0.0% (0)	0.8% (1)	0.0% (0)	0.0% (0)	0.0% (0)	1.0% (1)
Manager	6.2% (8)	12.2% (16)	7.7% (2)	23.1% (6)	5.8% (6)	9.5% (10)
Professional	63.1% (82)	60.3% (79)	61.5% (16)	42.3% (11)	63.5% (66)	64.8% (68)
Sales worker	3.8% (5)	1.5% (2)	3.8% (1)	3.8% (1)	3.8% (4)	1.0% (1)
Student	10.8% (14)	7.6% (10)	3.8% (1)	3.8% (1)	12.5% (13)	8.6% (9)
Technician and trades worker	3.1% (4)	5.3% (7)	7.7% (2)	11.5% (3)	1.9% (2)	3.8% (4)
Employment status (n=267)*						
Full time	73.5% (97)	81.5% (110)	76.9% (20)	92.6% (25)	72.6% (77)	78.7% (85)
Part time	18.2% (24)	6.7% (9)	23.1% (6)	3.7% (1)	17.0% (18)	7.4% (8)
Casual	6.8% (9)	7.4% (10)	0.0% (0)	3.7% (1)	8.5% (9)	8.3% (9)
Not applicable	1.5% (2)	4.4% (6)	0.0% (0)	0.0% (0)	1.9% (2)	5.6% (6)

* Missing data: body mass and body mass index self-reported: n=2 included surgery group missing (1 female, 1 male). Birth country: n=1 surgery excluded prior to baseline (male) missing. Indigenous: n=1 included control (female), n=2 control excluded prior to baseline (female), n=2 surgery excluded prior to baseline (male), n=1 surgery included (female) missing. Education: n=1 control excluded prior to baseline (female), n=1 surgery excluded prior to baseline (male) missing. Occupation: n=2 control included (1 female, 1 male), n=1 control excluded prior to baseline (female), n=1 surgery excluded prior to baseline (male), n=3 surgery included (female) missing. Employment: n=1 control excluded prior to baseline (female) missing.

The median age at enrolment was 32 years for the control group and 34 years for the surgery group; height and body mass were similar across groups. There was an even distribution of females and males across all participants (48% and 52%, respectively). Most participants were born in Australia, held a bachelor’s or higher degree and were employed full-time in professional roles. Overall, the cohort reflected a well-educated, employed population, predominantly engaged in professional occupations. A detailed dataset split for females/males can be found in online supplemental file 4.

#### Types of surgery

We used the Orchard Sports Injury Classification System,^24^ with additional information sought when needed, to record the type of surgery of the 135 participants enrolled in the surgery group (see online supplemental file 1). We grouped the participants into three main surgical categories (1) ACLR (n=81); (2) meniscus and/or cartilage surgery (n=45); (3) other (eg, loose intra-articular body removal, patellar tendon repair, medial or lateral collateral ligament reconstruction) (n=9). For participants with a history of more than one unilateral surgery or bilateral surgery, we defined a reference surgery to be used in subsequent studies using the following logic: reference surgery=the major surgery in the knee (ACL reconstruction>meniscus and/or cartilage surgery>other). When participants had undergone the same surgery on both knees, we defined the reference surgery as the most recent. Detailed data on types of surgeries are reported in table 3. For the control group, the reference knee was randomly chosen prior to baseline data collection.

**Table 3:** Details on the type of surgery and time since surgery provided at recruitment (telephone screening)

Variable	Enrolled (n=135)	Excluded prior to baseline (n=27)	Completed baseline testing (n=108)
Type of surgery			
ACLR	60.0% (81)	59.3% (16)	60.2% (65)
Meniscus and/or cartilage	33.3% (45)	37.0% (10)	32.4% (35)
Other	6.7% (9)	3.7% (1)	7.4% (8)
Time since reference surgery (years)	10.0±7.8 Median 8.0 (IQR 3.7 to 14.7)	8.3±6.7 Median 4.9 (IQR 2.7 to 14.4)	10.5±8.0 Median 8.3 (IQR 4.4 to 15.0)
Time since most recent surgery (years)	8.1±7.0 Median 5.4 (IQR 2.6 to 12.0)	7.2±6.5 Median 4.0 (IQR 2.5 to 11.4)	8.3±7.2 Median 5.5 (IQR 3.0 to 12.5)
Side of surgery			
Bilateral	13.3% (18)	18.5% (5)	12.0% (13)
Left	35.6% (48)	18.5% (5)	39.8% (43)
Right	51.1% (69)	63.0% (17)	48.1% (52)
Reference knee number of surgeries			
1	77.8% (105)	88.9% (24)	75.0% (81)
2	13.3% (18)	11.1% (3)	13.9% (15)
3	5.9% (8)	0.0% (0)	7.4% (8)
4	2.2% (3)	0.0% (0)	2.8% (3)
5	0.7% (1)	0.0% (0)	0.9% (1)
Non-reference knee number of surgeries			
0	86.7% (117)	81.5% (22)	88.0% (95)
1	10.4% (14)	14.8% (4)	9.3% (10)
2	3.0% (4)	3.7% (1)	2.8% (3)

ACLR, anterior cruciate ligament reconstruction.

#### Running-related characteristics

Running-related characteristics of the 268 participants included at the enrolment time-point are presented in the following sections.

##### Training load data (wearable technology—smartwatch)

At the enrolment time-point, 265 participants reported wearing a smartwatch (99%). Six different smartwatch devices were reported: Garmin 214 (81%); Apple 22 (8%); other 29 (11%), including Suunto, FitBit, Coros, Samsung and Polar. The online database used by our study (Smartabase, Fusion Sport Pty Ltd, Australia) had access to the application programming interface of a limited number of devices (eg, Garmin, Apple). Therefore, training load data were collected from 248/268 participants (92.5%) since the enrolment time point with participants’ consent.

##### Medication use for knee pain

Table 4 presents the self-reported use of any medication for knee pain reported by participants for the 3 months prior to enrolment time-point. Non-steroidal anti-inflammatory drugs (NSAIDs) were the most used medications across all groups, with a higher number of users observed in the surgery group compared with the control group, particularly among those who completed baseline testing (20.4% vs 10.4%). Topical NSAID creams and paracetamol were also frequently used. Codeine, tramadol and stronger opioids (eg, morphine) were rarely used. Cannabis use for knee pain was minimal, with only one participant (0.7%) in the surgery group reporting its use.

**Table 4:** Self-reported medication use for knee pain 3 months prior to enrolment time point

Drug	Enrolled (n=268)		Excluded prior baseline (n=54)		Completed baseline testing (n=214)	
	Control n=133	Surgery n=135	Control n=27	Surgery n=27	Control n=106	Surgery n=108
NSAID	12.0% (16)	18.5% (25)	18.5% (5)	11.1% (3)	10.4% (11)	20.4% (22)
Topical NSAID cream	6.0% (8)	11.1% (15)	7.4% (2)	11.1% (3)	5.7% (6)	11.1% (12)
Paracetamol	3.8% (5)	8.9% (12)	3.7% (1)	7.4% (2)	3.8% (4)	9.3% (10)
Codeine	0.0% (0)	2.2% (3)	0.0% (0)	0.0% (0)	0.0% (0)	2.8% (3)
Cannabis	0.0% (0)	0.7% (1)	0.0% (0)	0.0% (0)	0.0% (0)	0.9% (1)
Other	0.0% (0)	1.5% (2)	0.0% (0)	3.7% (1)	0.0% (0)	0.9% (1)
Tramadol	0.0% (0)	1.5% (2)	0.0% (0)	0.0% (0)	0.0% (0)	1.9% (2)
Morphine or other opioids	0.0% (0)	0.7% (1)	0.0% (0)	0.0% (0)	0.0% (0)	0.9% (1)

NSAID, non-steroidal anti-inflammatory drug.

##### Running behaviour (self-reported)

Online supplemental file 5 presents characteristics related to participants’ running behaviour, including running history, training practices and participation in sports other than running at the enrolment time-point. Participants in the surgery group consistently reported higher participation in weight-bearing sports other than running (31.6%) compared to the control group (16.8%). Engagement in non-weight-bearing sports was also more frequent in the surgery group (50.4%) than in the control group (43.2%).

Most participants began running regularly between the ages of 10 and 29 years (76% control group, 68% surgical group), with around 28% in both groups running for over 10 years. Weekly running distances were distributed similarly across groups, a higher percentage of the control group reported distances between 50 and 100 km per week (38%) and a higher percentage of the surgery group reported distances between 30 and 49 km per week (26%).

A higher percentage of the control group (58.6%) reported participating in running clubs compared with the surgical group (37.8%). Similarly, more controls engaged in structured training programmes (40.6%) compared with the surgical group (25.2%). Both groups presented similar levels of participation in upper-limb, core and lower-limb strength training.

### Baseline time-point

For the 214 who attended the face-to-face baseline time-point, a description of demographics and clinical characteristics collected in the university gait laboratory can be found in table 5. Participants’ median time between enrolment and baseline laboratory testing was 33 weeks with an IQR of 18 to 86 weeks. The reference knee had an even distribution across the right and left sides. Height, body mass and body mass index were comparable between groups, with males generally taller and heavier than females.

**Table 5:** Anthropometric and clinical details at the baseline time-point (participants who remained in the cohort n=214)

Variables	Control			Surgery		
	Female	Male	Combined	Female	Male	Combined
	N=54	N=52	N=106	N=53	N=55	N=108
Time between enrolment and lab test (weeks)	53.4±41.9 Median 35.5 (IQR 16.5 to 89.0)	62.3±39.3 Median 43.6 (IQR 29.4 to 98.6)	57.8±40.7 Median 37.4 (IQR 21.9 to 97.1)	35.9±38.1 Median 16.3 (IQR 8.5 to 41.4)	49.6±34.5 Median 33.0 (IQR 23.2 to 80.0)	42.9±36.8 Median 29.8 (IQR 15.2 to 76.3)
Reference knee (right)	51.9% (28)	57.7% (30)	54.7% (58)	47.2% (25)	56.4% (31)	51.9% (56)
Bilateral surgery	NA	NA	NA	17.0% (9)	7.3% (4)	12.0% (13)
Height (m)	1.67±0.05 Median 1.67 (IQR 1.64 to 1.72)	1.82±0.07 Median 1.82 (IQR 1.76 to 1.86)	1.75±0.09 Median 1.73 (IQR 1.67 to 1.82)	1.69±0.07 Median 1.69 (IQR 1.66 to 1.74)	1.81±0.06 Median 1.81 (IQR 1.77 to 1.85)	1.75±0.09 Median 1.75 (IQR 1.69 to 1.83)
Body mass (kg)	62.6±7.8 Median 61.0 (IQR 57.4 to 68.4)	77.0±10.5 Median 75.6 (IQR 71.4 to 83.8)	69.7±11.6 Median 69.1 (IQR 59.8 to 76.0)	64.4±10.0 Median 64.6 (IQR 57.9 to 70.0)	78.9±8.3 Median 79.6 (IQR 73.8 to 83.6)	71.8±11.7 Median 72.6 (IQR 64.3 to 80.9)
BMI (kg/m^2^)	22.3±2.4 Median 22.2 (IQR 20.4 to 23.8)	23.2±2.3 Median 22.8 (IQR 21.6 to 24.3)	22.7±2.4 Median 22.7 (IQR 20.8 to 24.2)	22.6±2.7 Median 22.0 (IQR 21.1 to 23.8)	24.1±2.2 Median 24.0 (IQR 22.9 to 25.5)	23.4±2.6 Median 23.3 (IQR 21.7 to 25.1)
Waist circumference (cm)	76.7±6.7 Median 76.0 (IQR 73.0 to 79.5)	85.9±7.4 Median 84.0 (IQR 80.0 to 89.5)	81.2±8.4 Median 80.0 (IQR 75.3 to 85.9)	78.9±8.4 Median 78.0 (IQR 73.0 to 81.5)	87.5±6.4 Median 87.0 (IQR 83.0 to 92.5)	83.2±8.6 Median 83.0 (IQR 77.8 to 89.6)
Hip circumference (cm)	96.5±7.2 Median 95.0 (IQR 92.0 to 99.7)	99.4±5.7 Median 99.0 (IQR 95.3 to 102.0)	97.9±6.6 Median 97.0 (IQR 93.2 to 101.0)	97.6±7.4 Median 98.0 (IQR 93.0 to 101.0)	99.9±4.7 Median 101.0 (IQR 97.0 to 103.7)	98.8±6.3 Median 99.7 (IQR 94.5 to 103.1)
Limb dominance (right)	88.9% (48)	94.2% (49)	91.5% (97)	90.6% (48)	89.1% (49)	89.8% (97)
Reference knee flexion active ROM (°)	142.0±4.1 Median 140.5 (IQR 140.0 to 145.0)	138.9±5.2 Median 140.0 (IQR 135.0 to 140.0)	140.5±4.9 Median 140.0 (IQR 138.0 to 144.0)	137.1±6.8 Median 138.0 (IQR 131.0 to 140.0)	133.3±7.0 Median 133.0 (IQR 130.0 to 140.0)	135.2±7.2 Median 135.0 (IQR 130.0 to 140.0)
Non-reference knee flexion active ROM (°)	141.3±4.3 Median 140.00 (IQR 140.0 to 145.0)	138.8±4.9 Median 140.0 (IQR 135.0 to 140.2)	140.1±4.8 Median 140.0 (IQR 138.0 to 143.0)	139.1±5.8 Median 140.0 (IQR 135.0 to 142.0)	136.2±6.8 Median 138.0 (IQR 134.0 to 140.0)	137.6±6.5 Median 140.0 (IQR 135.0 to 140.0)
Knee flexion ROM LSI* (%)	100.5±1.6 Median 100.0 (IQR 100.0 to 100.7)	100.0±1.6 Median 100.0 (IQR 100.0 to 100.0)	100.2±1.6 Median 100.0 (IQR 100.0 to 100.0)	98.2±2.8 Median 98.9 (IQR 96.6 to 100.0)	97.7±3.6 Median 100.0 (IQR 96.3 to 100.0)	97.9±3.2 Median 99.2 (IQR 96.4 to 100.0)
Knee extension passive ROM (cm†)	0.13±1.28 Median 0.00 (IQR −0.50 to 1.00)	−0.01±1.44 Median 0.00 (IQR −1.00 to 0.50)	0.06±1.36 Median 0.00 (IQR −0.88 to 0.88)	0.32±1.79 Median 0.00 (IQR −0.50 to 1.00)	−0.03±2.33 Median 0.00 (IQR −1.25 to 1.00)	0.14±2.08 Median 0.00 (IQR −1.00 to 1.00)
Reference knee medial joint palpation pain	3.7% (2)	0.0% (0)	1.9% (2)	15.1% (8)	7.3% (4)	11.1% (12)
Non-reference knee medial joint palpation pain	0.0% (0)	1.9% (1)	0.9% (1)	5.7% (3)	3.6% (2)	4.6% (5)
Reference knee lateral joint palpation pain	0.0% (0)	1.9% (1)	0.9% (1)	13.2% (7)	3.6% (2)	8.3% (9)
Non-reference knee lateral joint palpation pain	0.0% (0)	0.0% (0)	0.0% (0)	5.7% (3)	0.0% (0)	2.8% (3)

The reference knee has been previously described under the Types of surgery section.

* For LSI calculations, participants with bilateral surgery were excluded from summaries.

† Difference between reference and non-reference (positive values indicate a lower range of motion on the reference limb).

°, degree; BMI, body mass index; LSI, Limb Symmetry Index; NA, not applicable; ROM, range of motion.

Active knee range of motion was similar between the reference and non-reference knees, with a slight reduction in flexion range of motion in the surgery group. Knee extension passive range of motion was close to neutral across all groups. Pain on joint palpation was more frequently reported in the surgery group, particularly in the medial and lateral compartments of reference knees. Overall, the groups demonstrated similar baseline physical characteristics and functional measures with no apparent differences between females and males.

#### Training load data (wearable technology—smartwatch)

Smartwatch data were collected for 200/214 participants from the baseline time-point onwards (93.5%).

## Strengths and limitations

A key strength of the TRAIL study is its comprehensive, multi‐modal approach to characterising knee health among runners with and without heightened osteoarthritis risk. TRAIL integrates patient-reported outcomes, knee MRI, three-dimensional running biomechanics, performance-based function, clinical examination and objectively recorded training load data via wearable devices. Over a planned 10-year follow-up, this extensive dataset creates a robust framework for investigating the long-term relationship between running and knee health. In comparison to other longitudinal running cohorts globally, such as the National Runners’ Health Study in North America,^25^ RUNSAFE (worldwide)^26 27^ and SAFER (South Africa)^28^ cohorts examining running-related injuries and knee health—TRAIL provides a more complete dataset. Whereas many earlier studies were limited by the shorter follow-up and lack of biomechanical assessments or structural outcomes (MRI),^27^,^29^ TRAIL’s integration of daily running activity (wearable technology) with imaging and biomechanical testing over 10 years allows for a comprehensive exploration of the association between running exposure, joint mechanics and osteoarthritis risk.

Another strength of TRAIL is the implementation of adaptive recruitment strategies, which have resulted in a balanced representation of females and males across both the surgical and non-surgical control groups. This strength is particularly important as females represent only 10% of participants included in original sport and exercise medicine studies.^23^ We encourage other studies in the field to take a similar approach. Additional strengths include targeted recruitment from diverse sources (eg, running podcasts, clubs and social media), which ensures the inclusion of a ‘real-world’ cohort of runners aged 18–50 years.

Despite these strengths, several limitations warrant consideration. The absence of a non-runner control group may restrict the external validity of findings when generalising to the broader population and limit our ability to answer the question, “Is running bad for your knees?”. A large variability in the interval between enrolment and baseline laboratory testing due to COVID-19 pandemic lockdowns (median of 33 weeks) could introduce some level of heterogeneity in baseline measures. Of note, although all participants met the ≥3 runs/week on average in the previous 6 months eligibility criterion at recruitment, some reported lower running frequency in the previous month at enrolment. This is likely due to natural fluctuations in running habits that may occur due to injury, illness, competing life demands or seasonal variation. These variations reflect real-world running behaviour and will be accounted for in analyses using objectively measured running exposure from wearable devices. Additionally, reliance on self-reported data for certain aspects of running behaviour may be susceptible to recall bias. External factors such as COVID-19-related disruptions during recruitment and data collection might have impacted participant retention, running behaviour and self-reported measures. Within the surgery group, risk profiles for knee osteoarthritis may differ across subgroups. While our cohort profile does not explore subgroup-specific outcomes, the TRAIL dataset is well-positioned to explore these differences longitudinally, which may inform tailored prevention and management strategies based on individual surgery profiles.

### Collaboration

We welcome potential collaboration with other researchers. Researchers can visit the TRAIL website (https://trail.latrobe.edu.au/) for more information and submit queries regarding collaboration via the Contact Us web page.

## Supplementary material

10.1136/bmjopen-2025-101625 online supplemental file 1

10.1136/bmjopen-2025-101625 online supplemental file 2

10.1136/bmjopen-2025-101625 online supplemental file 3

10.1136/bmjopen-2025-101625 online supplemental file 4

10.1136/bmjopen-2025-101625 online supplemental file 5

## Data Availability

Data are available upon reasonable request.
