# Supplementary material for: Cohort profile: trajectory of knee health in runners with and without heightened osteoarthritis risk (TRAIL) in Australia—prospective cohort study
Source: BMJ Open. 2025 Sep 28;15(9):e101625. doi: 10.1136/bmjopen-2025-101625 (PMC12481359; doi:10.1136/bmjopen-2025-101625)
Supplement: online supplemental file 1 [file bmjopen-15-9-s001.docx]



**Phone Screening**

**Participant Details**

**Phone Number**

**Email**

**Research Participant Number**

**Sex**

**Age**

**Date of Birth**

**Q1**

Thank you for your interest in the Trail Study. Today we will be going through some further screening questions to make sure you are eligible for the study.

We would like to start by conﬁrming your eligibility for the study by asking you a couple of questions about your running history and your ability to participate in the study.

Over the last 6 months, in an average week, how many times do you run?

3 or more

Less than 3 *(ineligible and stop screening)*

**Q2**

Over the last 6 months, in an average week, how many kilometres do you accumulate per week?

10 or more

Less than 10 *(ineligible and stop screening)*

**Postcode**

**Address**

**Q3**

To have an MRI, you cannot have any metal in your vital organs. Do you have a pace-maker, metal implants or metal in your eyes or any other reasons you can't have an MRI (claustrophobic)?

Yes *(ineligible and stop screening)*

No

**Q4**

Do you consent to having an MRI of your knee over 2 time points (baseline, 4 years and 10 years)?

Yes

No *(ineligible and stop screening)*

**Q5**

Are you currently pregnant?

Yes *(ineligible and stop screening)*

No

**Q6**

Have you had any ankle, hip or other lower-limb surgery in the past? (excluding knee surgery)

Yes *(ineligible and stop screening)*

No

**Q7**

Have you had any knee surgery?

Yes

No

If ***no*** to Q7 (control group participant),

**Q8**

Do you have a history of traumatic knee injury (requiring period not being able to put weight on that leg) for >24 hours

Yes

No *(ineligible and stop screening)*

If ***yes*** to Q7 (surgical group participant),

**Q9**

Was this surgery due to a fracture in your knee? Have you had an osteotomy or joint replacement surgery?

Yes *(ineligible and stop screening)*

No

**Knee Surgical History**

We would now like to discuss the previous surgical and injury history of both knees. We will start with your left knee ﬁrst.

| **Surgery Number** | **Surgery Date** | **Laterality (Surgery)** | **OSICS Surgery** | **Days Since Knee Surgery** | **Years Since Knee Surgery** |  |
| --- | --- | --- | --- | --- | --- | --- |
| 1 |  |  |  |  |  |  |

**Add Row**

**Knee Injury History (all participants)**

We would now like to discuss the injury history of both knees. We will start with your left knee ﬁrst.

| **Knee Injury Number** | **Laterality (Knee Injury)** | **Knee OSICS Injury** | **Knee MOI** | **Knee Grindem** | **Knee Injury Date** |  |
| --- | --- | --- | --- | --- | --- | --- |
| 1 |  |  |  |  |  |  |

**Add Row**

**Lower Limb Injury History (all participants)**

Now we need to ask you about other injuries you may have had to the other joints in your lower limbs. Have you had any other lower limb injuries? (to your ankle, hip or other structures excluding your knee)

| **LL Injury Number** | **Laterality (LL Injury)** | **LL Osics Injury** | **LL MOI** | **LL Grindem** | **LL Injury Date** |  |
| --- | --- | --- | --- | --- | --- | --- |
| 1 |  |  |  |  |  |  |

**Add Row**

**Q10**

What do you usually use to record your runs?

Garmin Polar Strava Other

**Q12**

Where did you hear about the TRAIL running study?

TRAIL Website Instagram Facebook Twitter

Inside running podcast

**Q13**

Which social media platform do you engage with the most?

Instagram

Facebook

Twitter

**Q14**

Would you be part of a Facebook TRAIL running study group to access content?

Yes

No
