# Supplementary material for: Cohort profile: trajectory of knee health in runners with and without heightened osteoarthritis risk (TRAIL) in Australia—prospective cohort study
Source: BMJ Open. 2025 Sep 28;15(9):e101625. doi: 10.1136/bmjopen-2025-101625 (PMC12481359; doi:10.1136/bmjopen-2025-101625)
Supplement: online supplemental file 2 [file bmjopen-15-9-s002.docx]

**Enrolment time point: Socio-demographic factors and running-related questions**

**Demographic data**



**Height**

(Metres)

**Weight**

(kg)

**Country of Birth**

**Current Postcode**

**Aboriginal or Torres Strait Islander origin**

No

Yes, Aboriginal

Yes, Torres Strait Islander

Yes, both Aboriginal and Torres Strait Islander

**Highest level of education** Never attended school Some primary school Completed primary school

Some high school (i.e. year7-year11, form 1-5) High school completion (i.e. year 12, form 6, HSC) Graduate diploma

Bachelor's degree Master's degree Doctorate degree

**Occupation**

(put student if primarily studying, or on leave, retired or unemployed etc. if not working)

**Current employment status** Casual Part time Full time

Not applicable

(select all that apply)

**Family history of Osetoarthritis**

Does any of your family members have a history of being diagnosed with osteoarthritis or having surgery for osteoarthritis?

Yes

No

**If yes, which joint?**

**Running History**

**Other weight-bearing sport participation**

Over the last month, have you participated in other weight-bearing sports (apart from running) on average at least once per week?

Yes, I participate in other weight-bearing sport besides running (e.g. soccer, basketball, football, tennis, etc.) No, I do not play in other weight-bearing sport other than running

**Weight-bearing sports played**

Please list each sport other than running you currently participate in.

**Weight-bearing Sport Frequency**

On average, how frequently do you participate in these sports each week?

1

2

3

4

5

6

7

**Other non-weight-bearing sport participation**

Over the last month, have you participated in other non-weight-bearing sports (apart from running) on average at least once per week?

Yes, I participate in other non-weight-bearing sport besides running (e.g. cycling, swimming etc) No, I do not play in other non-weight-bearing sport other than running

**Non-weight-bearing sports played**

Please list each sport other than running you currently participate in.

**Non-weight-bearing Sport Frequency**

On average, how frequently do you participate in these sports each week?

1

2

3

4

5

6

7

**Age started running**

How old were you when you started to run on a regular basis (more than 3 times per week)?

Below 10

Between 10 - 14

Between 15 - 19

Between 20 - 24

Between 25 - 29

Between 30 - 34

Between 35 - 39

Between 40 - 44

Between 45 - 50

Over 50

**Years Running Regularly**

How many years have you been running regularly (more than 3 times per week) Less than 1 year

Between 1 - 3 years Between 3 - 5 years Between 5 - 10 years Between 10 - 20 years More than 20 years

**Average Distance Per Week**

Over the last month, on average, how many kilometres have you run per week

Between 10 - 19 km Between 20 - 29 km Between 30 - 49 km Between 50 - 100 km More than 100 km

**Longest Run**

What was the longest distance you have ever ran in one day/session?

Less than 10 km

Between 10 - 21 km

Half-marathon (21.1 km) Between 21.2 - 30 km Between 31 - 42.1 km Marathon (42.2 km)

Ultra-marathon (over 42.2 km)

**Number of Runs per Week**

Over the last month, on average, how many times have you ran per week?

`

1 2 3 4 5 6 7 8 9 10+

**Running Club**

Are you currently part of a running club or group (more than 3 people)?

Yes

No

**Interval Training**

Over the last month, on average, how many times per week did you run speed intervals? (Training session involving bursts or running at signiﬁcantly higher speed than your comfortable running speed)

Never

Less than once per week

Once per week

Twice per week

More than twice per week

**Highest Weekly Distance**

What is your highest total weekly running distance ever?

Between 5 - 15 km Between 15 - 25 km Between 25 - 35 km Between 35 - 45 km Between 45 - 55 km Between 55 - 65 km Between 65 - 75 km Between 75 - 85 km Between 85 - 95 km Between 95 - 105 km More than 105 km

I don't know

**5K PB**

What is your fastest ever 5 kilometer

Less than 14 min Between 14 - 17 min Between 17 - 20 min Between 20 - 23 min Between 23 - 26 min Between 26 - 29 min Between 29 - 31 min Between 31 - 34 min Between 34 - 37 min Between 37 - 40 min More than 40 min

I don't know

**Running Program**

Over the last month, how did you structure your running program?

I don't follow a running program

I follow a running program from a coach or a health professional

I follow a running program from a newspaper, magazine of the internet

I follow a running program from a friend or relative

I do my own running program

I only follow a running program when I am preparing for a running event (i. e. marathon, half-marathon)

**Upper body training**

Over the last month, have you done any upper limb (arm) strength training?

Yes

No

**Upper body training frequency**

On average, over the past month, how many days per week have you done upper limb (arm) strength training?

1

2

3

4

5

6

7

**Lower body training**

Over the last month, have you done any lower limb (leg) strength training?

Yes

No

**Lower body training frequency**

On average, over the past month, how many days per week have you done lower limb (leg) strength training?

1

2

3

4

5

6

7

**Core Training**

Over the last month, have you done any core/trunk strength training?

Yes

No

**Core training frequency**

On average, over the past month, how many days per week have you done core/trunk strength training?

1

2

3

4

5

6

7

**Stretching**

Over the last month, have you done any upper or lower body stretching?

Yes

No

**Stretching frequency**

On average, over the past month, how many days per week have you done upper or lower body stretching?

1

2

3

4

5

6

7

**Training & COVID-19**

The following questions ask about changes to your running behavior due to COVID-19 restrictions

**COVID Training frequency**

As a result of the COVID-19 restrictions in 2020, did your weekly running volume change?

Increased the number of weekly sessions and/or distance (km) by more than 10% Decreased the number of weekly sessions and/or distance (km) by more than 10% No change in the weekly training

**Knee Symptoms**

The following questions ask you about your current knee symptoms

**Running Knee Pain**

During the last week, what was your average level of knee pain during running?

`

0 - No pain

**.**

100 - Worst Pain

**Left Knee Swelling**

Over the last week, during or after running did you experience any swelling in your LEFT KNEE?

Yes No

**Right Knee Swelling**

Over the last week, during or after running did you experience any swelling in your RIGHT KNEE?

Yes No

**Left Knee Stiffness**

Over the last week, did you experience any stiffness in your LEFT KNEE when you ﬁrst wake up in the morning?

Yes No

**Right Knee Stiffness**

Over the last week, did you experience any stiffness in your RIGHT KNEE when you ﬁrst wake up in the morning?

Yes No

**Left Knee Crepitus**

Over the last week, did you experience any crepitus (crack, grind, pop sounds) in your LEFT KNEE?

Yes No

**Right Knee Crepitus**

Over the last week, did you experience any crepitus (crack, grind, pop sounds) in your RIGHT KNEE?

Yes No

**Knee Imaging**

**X-ray**

Have you ever had an x-ray of your knee joint that described osteoarthritis?

Yes No

**MRI**

Have you ever had an MRI of your knee joint that described osteoarthritis?

Yes No

**Running and Osteoarthritis (OA)**

The following questions ask you about your beliefs regarding running and knee osteoarthritis

**Running & Knee Health**

In general, you see regular running as an activity that hurts the knee joint

I strongly disagree I disagree Uncertain I agree I strongly agree

**Frequency & OA**

Frequent running can lead to getting knee osteoarthritis

I strongly disagree I disagree Uncertain I agree I strongly agree

**Distance & OA**

Running long distances (such as >10 km, marathon distance) can lead to getting knee osteoarthritis

I strongly disagree I disagree Uncertain I agree I strongly agree

**OA & Continuing running**

People with knee osteoarthritis who continue to run will sustain greater knee cartilage damage leading to more severe osteoarthritis

I strongly disagree I disagree Uncertain I agree I strongly agree

**Running Vs Symptoms**

It is ﬁne for people who have knee osteoarthritis to run, as long as they don't have symptoms on the day they go running

I strongly disagree I disagree Uncertain I agree I strongly agree

**OA & Joint Replacement**

A person with knee osteoarthritis who keeps running regularly will speed up the need for joint replacement surgery

I strongly disagree I disagree Uncertain I agree I strongly agree

**Avoiding OA**

One of the reasons you don't run regularly is to avoid getting osteoarthritis in your knees

I strongly disagree I disagree Uncertain I agree I strongly agree

**Knee Health Advice**

If you have had knee pain, who has provided you with advice on running and knee joint health?

Family physician (GP) Rheumatologist

Sports medicine physician Orthopaedic surgeon Physiotherapist Chiropractor

Athletic therapist

Friend, colleague or family member

Public forums or presentations

Internet Television Radio

Print media (newspapers, magazines, books)

Scientiﬁc literature

Other

I haven't received any advice on running and knee joint health

**Knee Pain Behaviour**

If you were to develop knee pain (but without a diagnosis of knee osteoarthritis), you would.... (select all that apply)

Stop running

Running less frequently

Run less distance

Run slower

Not change my running habits

**OA Diagnosis**

Have you been diagnosed with knee osteoarthritis by a healthcare professional?

Yes

No

***If no,* Hypothetical Diagnosis Behaviour**

If you were diagnosed with knee osteoarthritis by a healthcare professional, you would...

Stop running

Running less frequently

Run less distance

Run slower

Not change my running habits

***If yes,*** **Pain Development Behaviour**

When you developed knee pain (but without knowing that you had osteoarthritis), you....

Stopped running temporarily

Decreased my running frequency (number of times per week)

Decreased my running distance

Decreased my running speed

Did not change my running habits

***If yes,*** **OA Diagnosis Behaviour**

When you were diagnosed with knee osteoarthritis, you....

Stopped running temporarily

Decreased my running frequency (number of times per week)

Decreased my running distance

Decreased my running speed

Did not change my running habits

**Medication**

The following question asks about medications that you have taken for your knee

Do you use, or have used, medication to help your knee feel better in the last 3 months?

**Knee Pain Medication** No, I don't take any medication for my knee

Paracetamol (e.g. Panadol, Panamax, Herron Paracetamol)

Non-steroidal anti-inﬂammatory drugs/NSAID (e.g. Ibuprofen, aspirin)

Topical NSAID cream (e.g. Voltaren cream/gel)

Glucosamine

Hyaluronic acid injection into your knee joint

Morphine or other opioids

Tramadol

Codeine (e.g. Panadeine) Antidepressants (speciﬁcally for

pain)

Anticonvulsants (speciﬁcally for pain) (e.g.

Gabapentin, Pregabalin, Lyrica) Methotrexate

Bisphosphonate (e.g. Fosamax, Actonel)

Herbal supplements

Cannabis

Other

**Menstrual Cycle (females only)**

The following questions ask about your menstrual cycle. We are asking these questions because female hormonal changes can effect ligament laxity and pain perceptions. Please if you feel uncomfortable completing these, select the option "I prefer not to answer".

**Age of Menarche**

What age were you when you had your ﬁrst period?

15 years old or younger

16 years old or older I don't remember Prefer not to answer

**Cycle Regularity**

Over the past year, how regular is your cycle?

Every 25-35 day (regular cycle)

Less than every 25 days (or irregular cycle) More than 35 days

I don't know

Other

Prefer not to answer

**Cycle Disruption**

Have your periods ever stopped for 3 consecutive months or longer (other than pregnancy)?

Yes

No

Prefer not to answer

**Training Cycle Disruption**

Do you experience any changes to your menstrual cycle when you change your training intensity or frequency?

Yes

No Unsure

Prefer not to answer

***If yes,* Cycle Changes**

How has your cycle changed in relation to your training? (select all that apply)

I have lighter menstruation

My cycle is shorter in duration

My cycle stops

My cycle is longer in duration

I have heavier menstruation

Other

Prefer not to answer

**Oral Contraception**

Do you use oral hormonal contraception?

Yes

No

Prefer not to answer

***If yes,* Contraception Reason**

Why do you use oral hormonal contraception? (select all that apply)

Contraception

Reduction of menstrual pain /

symptoms

Reduction of bleeding

To regulate the menstrual cycle to performance

Otherwise menstruation stops

Other

Prefer not to answer

**Running Shoes**

The following questions ask you about the shoes that you wear during running

**Running Shoes**

Which brand of running shoes do you primarily use? (Select all that apply) Adidas

Asics

Brooks

New Balance

Nike

Mizuno

Under Armour

Saucony

Other

**Shoe type**

Which type of running shoes do you currently use? (Select all that apply) Motion control

Neutral Maximal Minimal Other

I don't know

**Shoe Cushioning**

What is the cushioning level of your running shoes?

Highly cushioned running shoes Moderately cushioned running shoes Low-cushioned running shoes

I switch between running shoes with different levels of cushioning

I don't know

**Shoe Important Factors**

What do you consider important when buying running shoes? (Select all that apply)

A shoe that controls my foot motion/posture (pronation, supination, neutral)

A shoe that suits my foot-strike pattern (forefoot, midfoot, heel strike)

A shoe that is comfortable

That the price is low/reasonable

That the shoe is similar to my previous running shoes

That I get advice from running shoe retailer

That I get advice from healthcare professional (physiotherapist/podiatrist)

I get advice from a coach/trainer

The shoes aims to improve my performance/personal best

I like the design and colours

I chose by my favourite brand

I don't buy shoes, the are provided by my sponsor

I don't buy running shoes as I run barefoot

I don't know

**Running Accessories**

The following questions ask you about accessories you use when running

**Watch Frequency**

How often do you wear a watch to monitor your running activities?

All of my runs

More than 90% of my runs

50% - 90% of my runs

Less than 50% of my runs

I carry my phone to track my running instead of a watch

I don't know

**Supports & Braces**

Do you currently use one or more of the following items when running? (Select all that apply) Ankle brace

Knee brace

Tape to support my ankle Tape to support my knee Sports bra

Insoles (e.g. orthotics) Compression socks

Baby jogger or baby stroller

Backpack Hydration pack I don't know

Nutrition

No, I don't use any equipment listed above
