# Supplementary material for: Cohort profile: trajectory of knee health in runners with and without heightened osteoarthritis risk (TRAIL) in Australia—prospective cohort study
Source: BMJ Open. 2025 Sep 28;15(9):e101625. doi: 10.1136/bmjopen-2025-101625 (PMC12481359; doi:10.1136/bmjopen-2025-101625)
Supplement: online supplemental file 3 [file bmjopen-15-9-s003.docx]

Reference Knee =

**TRAIL laboratory-based baseline testing**

Date / /

| Subject Code: |  |
| --- | --- |

| Tested blinded: | Yes No |
| --- | --- |

Sex: M F D.O.B.:

| Dominant Limb: | L R |
| --- | --- |

Testers:

**PHYSICAL TESTING**

| **Patient data and measurements** | |  |
| --- | --- | --- |
| Height (cm) |  |  |
| Weight (kg) |  |  |
| Waist Circumference (Umbilical line) cm |  |  |
| Hip Circumference  (Widest point of greater trochanter) |  |  |
| Distal greater trochanter to lateral joint line knee (cm) | L __________ R __________ |  |
| Distal greater trochanter to distal tip of lateral tibia line (cm) | L __________ R __________ |  |

| **Clinical Assessment in supine** |
| --- |

| **Knee range of movement (ROM)**  **QUADS**   - **60 degrees**   - Force plate 5cm proximal to medial malleolus (ER) or lateral malleolus (IR)   - Subject instructed *“turn shin inwards towards centre (ER) or outward, keeping knees together (IR) as hard as possible”*   - Each performed 3 times, 1 sec build up, 3 secs as hard as possible: “go, go, go…”   - 5 secs between each repetition, 30 secs minimum between each test |
| --- |

***Flexion Active (supine active, goniometer)***

| LEFT | Trial 1 (°) | RIGHT | Trial 1 (°) |
| --- | --- | --- | --- |
|  |  |  |  |

- Subject supine ask them to actively bring their heel to their bum.

***Extension passive (prone heel height difference)***

- Subject prone with patella on very edge of plinth (make sure left and right patella distance from edge of plinth even, about 2 finger widths).
- Measure heel height difference with ruler from floor (cm)

|  | Trial 1 left | Trial 1 Right |  |  |
| --- | --- | --- | --- | --- |
| Heel height difference |  |  | More Flexed: Left / Right  Cm= |  |

**Knee Joint palpation**

| A) Medial knee joint palpation  (Participant reports pain on palpation) | **Left**  +ive -ive | **Right**  +ve -ve |  |
| --- | --- | --- | --- |
| B) Lateral knee joint palpation  (Participant reports pain on palpation) | **Left**  +ive -ive | **Right**  +ve -ve |  |
| A) Test for crepitus in standing  (Double leg squat x 2) palpate kneecap | **Left**  +ive -ive | **Right**  +ve -ve |  |

| **Patient-reported outcomes collected** | |
| --- | --- |
| Knee injury and Osteoarthritis Outcome Score (KOOS) |  |
| The medical examination sports injury surveillance questionnaire (SPEX) |  |
| Athlete Sleep Screening Questionnaire (ASSQ) |  |
| Tampa Scale for Kinesiophobia (TSK) |  |
| Knee-Self Efficacy Scale (K-SES) |  |
| Patient Acceptable Symptom State (PASS) |  |
| Achilles questionnaire (VISA-A) |  |
