# Supplementary material for: Cohort profile: trajectory of knee health in runners with and without heightened osteoarthritis risk (TRAIL) in Australia—prospective cohort study
Source: BMJ Open. 2025 Sep 28;15(9):e101625. doi: 10.1136/bmjopen-2025-101625 (PMC12481359; doi:10.1136/bmjopen-2025-101625)
Supplement: online supplemental file 5 [file bmjopen-15-9-s005.docx]

Supplementary File 5 – Self-reported running behaviour of participants at the enrolment timepoint.

| **Variable** | **Enrolled (n=268)** | | **Excluded prior baseline (n=54)** | | **Completed baseline testing (n=214)** | |
| --- | --- | --- | --- | --- | --- | --- |
|  | **Control** | **Surgery** | **Control** | **Surgery** | **Control** | **Surgery** |
|  | ***n=133*** | ***n=135*** | ***n=27*** | ***n=27*** | ***n=106*** | ***n=108*** |
| **Participation in other weight-bearing sport (n=264)*** | 16.8% (22) | 31.6% (42) | 18.5% (5) | 28.0% (7) | 16.3% (17) | 32.4% (35) |
| **Participation in other non-weight-bearing sport (n=265)*** | 43.2% (57) | 50.4% (67) | 51.9% (14) | 42.3% (11) | 41.0% (43) | 52.3% (56) |
| **Age started running regularly (n=267)*** | | | | | | |
| <10 years | 7.6% (10) | 6.7% (9) | 18.5% (5) | 3.7% (1) | 4.8% (5) | 7.4% (8) |
| 10 to 14 years | 18.2% (24) | 14.1% (19) | 11.1% (3) | 22.2% (6) | 20.0% (21) | 12.0% (13) |
| 15 to 19 | 15.9% (21) | 20.0% (27) | 18.5% (5) | 29.6% (8) | 15.2% (16) | 17.6% (19) |
| 20 to 24 | 18.2% (24) | 15.6% (21) | 22.2% (6) | 14.8% (4) | 17.1% (18) | 15.7% (17) |
| 25 to 29 | 24.2% (32) | 18.5% (25) | 22.2% (6) | 14.8% (4) | 24.8% (26) | 19.4% (21) |
| 30 to 34 | 12.1% (16) | 17.0% (23) | 7.4% (2) | 11.1% (3) | 13.3% (14) | 18.5% (20) |
| 35 to 39 | 3.0% (4) | 3.7% (5) | 0.0% (0) | 0.0% (0) | 3.8% (4) | 4.6% (5) |
| 40 to 44 | 0.8% (1) | 4.4% (6) | 0.0% (0) | 3.7% (1) | 1.0% (1) | 4.6% (5) |
| **Years running regularly** | | | | | | |
| <1 year | 3.0% (4) | 8.9% (12) | 3.7% (1) | 7.4% (2) | 2.8% (3) | 9.3% (10) |
| 1 to <3 years | 24.1% (32) | 19.3% (26) | 18.5% (5) | 22.2% (6) | 25.5% (27) | 18.5% (20) |
| 3 to <5 years | 23.3% (31) | 19.3% (26) | 22.2% (6) | 7.4% (2) | 23.6% (25) | 22.2% (24) |
| 5 to <10 years | 21.8% (29) | 23.0% (31) | 22.2% (6) | 18.5% (5) | 21.7% (23) | 24.1% (26) |
| 10 to <20 years | 24.1% (32) | 17.0% (23) | 25.9% (7) | 22.2% (6) | 23.6% (25) | 15.7% (17) |
| ≥20 years | 3.8% (5) | 12.6% (17) | 7.4% (2) | 22.2% (6) | 2.8% (3) | 10.2% (11) |
| **Average weekly running distance in past month (n=267)*** | | | | | | |
| 10 to 19 km | 13.5% (18) | 25.4% (34) | 18.5% (5) | 22.2% (6) | 12.3% (13) | 26.2% (28) |
| 20 to 29 km | 13.5% (18) | 22.4% (30) | 14.8% (4) | 18.5% (5) | 13.2% (14) | 23.4% (25) |
| 30 to 49 km | 28.6% (38) | 26.1% (35) | 25.9% (7) | 18.5% (5) | 29.2% (31) | 28.0% (30) |
| 50 to 100 km | 38.3% (51) | 23.1% (31) | 25.9% (7) | 33.3% (9) | 41.5% (44) | 20.6% (22) |
| >100 km | 6.0% (8) | 3.0% (4) | 14.8% (4) | 7.4% (2) | 3.8% (4) | 1.9% (2) |
| **Weekly running frequency in past month** | | | | | | |
| 1 | 1.5% (2) | 0.7% (1) | 0.0% (0) | 0.0% (0) | 1.9% (2) | 0.9% (1) |
| 2 | 0.0% (0) | 6.7% (9) | 0.0% (0) | 7.4% (2) | 0.0% (0) | 6.5% (7) |
| 3 | 22.6% (30) | 35.6% (48) | 14.8% (4) | 25.9% (7) | 24.5% (26) | 38.0% (41) |
| 4 | 24.8% (33) | 25.2% (34) | 25.9% (7) | 33.3% (9) | 24.5% (26) | 23.1% (25) |
| 5 | 21.8% (29) | 18.5% (25) | 29.6% (8) | 14.8% (4) | 19.8% (21) | 19.4% (21) |
| 6 | 14.3% (19) | 6.7% (9) | 11.1% (3) | 7.4% (2) | 15.1% (16) | 6.5% (7) |
| 7 | 10.5% (14) | 5.2% (7) | 7.4% (2) | 7.4% (2) | 11.3% (12) | 4.6% (5) |
| 8 | 3.0% (4) | 0.7% (1) | 7.4% (2) | 3.7% (1) | 1.9% (2) | 0.0% (0) |
| 9 | 0.0% (0) | 0.7% (1) | 0.0% (0) | 0.0% (0) | 0.0% (0) | 0.9% (1) |
| 10+ | 1.5% (2) | 0.0% (0) | 3.7% (1) | 0.0% (0) | 0.9% (1) | 0.0% (0) |
| **Frequency of running interval training in past month** | | | | | | |
| Never | 8.3% (11) | 18.5% (25) | 18.5% (5) | 7.4% (2) | 5.7% (6) | 21.3% (23) |
| Less than once per week | 12.0% (16) | 23.7% (32) | 0.0% (0) | 22.2% (6) | 15.1% (16) | 24.1% (26) |
| Once per week | 42.1% (56) | 33.3% (45) | 29.6% (8) | 37.0% (10) | 45.3% (48) | 32.4% (35) |
| Twice per week | 33.8% (45) | 21.5% (29) | 51.9% (14) | 29.6% (8) | 29.2% (31) | 19.4% (21) |
| More than twice per week | 3.8% (5) | 3.0% (4) | 0.0% (0) | 3.7% (1) | 4.7% (5) | 2.8% (3) |
| **Personal best time for 5km run** | | | | | | |
| <14 min | 0.8% (1) | 0.7% (1) | 0.0% (0) | 0.0% (0) | 0.9% (1) | 0.9% (1) |
| 14 to <17 min | 16.5% (22) | 9.6% (13) | 22.2% (6) | 14.8% (4) | 15.1% (16) | 8.3% (9) |
| 17 to <20 min | 36.1% (48) | 31.1% (42) | 25.9% (7) | 33.3% (9) | 38.7% (41) | 30.6% (33) |
| 20 to <23 min | 27.8% (37) | 24.4% (33) | 33.3% (9) | 22.2% (6) | 26.4% (28) | 25.0% (27) |
| 23 to <26 min | 15.8% (21) | 21.5% (29) | 14.8% (4) | 11.1% (3) | 16.0% (17) | 24.1% (26) |
| 26 to <29 min | 1.5% (2) | 6.7% (9) | 0.0% (0) | 7.4% (2) | 1.9% (2) | 6.5% (7) |
| 29 to <31 min | 0.8% (1) | 3.0% (4) | 3.7% (1) | 11.1% (3) | 0.0% (0) | 0.9% (1) |
| I don't know | 0.8% (1) | 3.0% (4) | 0.0% (0) | 0.0% (0) | 0.9% (1) | 3.7% (4) |
| **Highest weekly total running volume ever** | | | | | | |
| 5 to <15 km | 2.3% (3) | 0.7% (1) | 3.7% (1) | 0.0% (0) | 1.9% (2) | 0.9% (1) |
| 15 to <25 km | 3.0% (4) | 5.2% (7) | 3.7% (1) | 11.1% (3) | 2.8% (3) | 3.7% (4) |
| 25 to <35 km | 6.0% (8) | 11.9% (16) | 7.4% (2) | 14.8% (4) | 5.7% (6) | 11.1% (12) |
| 35 to <45 km | 6.8% (9) | 8.1% (11) | 3.7% (1) | 3.7% (1) | 7.5% (8) | 9.3% (10) |
| 45 to <55 km | 7.5% (10) | 11.9% (16) | 3.7% (1) | 7.4% (2) | 8.5% (9) | 13.0% (14) |
| 55 to <65 km | 11.3% (15) | 10.4% (14) | 7.4% (2) | 14.8% (4) | 12.3% (13) | 9.3% (10) |
| 65 to <75 km | 11.3% (15) | 14.8% (20) | 11.1% (3) | 7.4% (2) | 11.3% (12) | 16.7% (18) |
| 75 to <85 km | 14.3% (19) | 6.7% (9) | 3.7% (1) | 3.7% (1) | 17.0% (18) | 7.4% (8) |
| 85 to <95 km | 1.5% (2) | 8.1% (11) | 0.0% (0) | 7.4% (2) | 1.9% (2) | 8.3% (9) |
| 95 to <105 km | 9.8% (13) | 4.4% (6) | 7.4% (2) | 3.7% (1) | 10.4% (11) | 4.6% (5) |
| ≥105 km | 24.8% (33) | 16.3% (22) | 40.7% (11) | 25.9% (7) | 20.8% (22) | 13.9% (15) |
| I don't know | 1.5% (2) | 1.5% (2) | 7.4% (2) | 0.0% (0) | 0.0% (0) | 1.9% (2) |
| **Longest distance ever run (n=267)*** | | | | | | |
| <10 km | 1.5% (2) | 0.0% (0) | 3.8% (1) | 0.0% (0) | 0.9% (1) | 0.0% (0) |
| 10 to 21 km | 12.9% (17) | 20.0% (27) | 7.7% (2) | 22.2% (6) | 14.2% (15) | 19.4% (21) |
| Half-marathon (21.1 km) | 10.6% (14) | 13.3% (18) | 15.4% (4) | 3.7% (1) | 9.4% (10) | 15.7% (17) |
| 21.2 to 30 km | 17.4% (23) | 14.8% (20) | 3.8% (1) | 14.8% (4) | 20.8% (22) | 14.8% (16) |
| 31 to 42.1 km | 6.1% (8) | 5.2% (7) | 11.5% (3) | 14.8% (4) | 4.7% (5) | 2.8% (3) |
| Marathon (42.2 km) | 30.3% (40) | 26.7% (36) | 15.4% (4) | 22.2% (6) | 34.0% (36) | 27.8% (30) |
| Ultra-marathon (over 42.2 km) | 21.2% (28) | 20.0% (27) | 42.3% (11) | 22.2% (6) | 16.0% (17) | 19.4% (21) |
| **Structure of training program in the last month** | | | | | | |
| I do my own running program | 31.6% (42) | 32.6% (44) | 37.0% (10) | 48.1% (13) | 30.2% (32) | 28.7% (31) |
| I don't follow a running program | 9.0% (12) | 20.0% (27) | 7.4% (2) | 11.1% (3) | 9.4% (10) | 22.2% (24) |
| I follow a running program from a coach or a health professional | 40.6% (54) | 25.2% (34) | 37.0% (10) | 29.6% (8) | 41.5% (44) | 24.1% (26) |
| I follow a running program from a friend or relative | 3.0% (4) | 3.7% (5) | 3.7% (1) | 3.7% (1) | 2.8% (3) | 3.7% (4) |
| I follow a running program from a newspaper, magazine from the internet | 3.8% (5) | 3.0% (4) | 3.7% (1) | 3.7% (1) | 3.8% (4) | 2.8% (3) |
| I only follow a running program when I am preparing for a running event (e.g., marathon, half-marathon) | 12.0% (16) | 15.6% (21) | 11.1% (3) | 3.7% (1) | 12.3% (13) | 18.5% (20) |
| **Member of running club** | 58.6% (78) | 37.8% (51) | 44.4% (12) | 37.0% (10) | 62.3% (66) | 38.0% (41) |
| **Additional training in the last month (At least 1 session)** | | | | | | |
| Upper-limb strength training (n=267)* | 64.7% (86) | 73.9% (99) | 44.4% (12) | 66.7% (18) | 69.8% (74) | 75.7% (81) |
| Lower-limb strength training (n=267)* | 75.8% (100) | 77.8% (105) | 66.7% (18) | 74.1% (20) | 78.1% (82) | 78.7% (85) |
| Core strength training (n=266)* | 78.9% (105) | 82.0% (109) | 74.1% (20) | 61.5% (16) | 80.2% (85) | 86.9% (93) |
| Upper/lower-limb stretching (n=267)* | 81.2% (108) | 75.4% (101) | 77.8% (21) | 77.8% (21) | 82.1% (87) | 74.8% (80) |
| **Shoe brand preference (multiple selections possible) (n=267)*** | | | | | | |
| Nike | 60.6% (80) | 39.3% (53) | 55.6% (15) | 44.4% (12) | 61.9% (65) | 38.0% (41) |
| Asics | 25% (33) | 33.3% (45) | 22.2% (6) | 40.7% (11) | 25.7% (27) | 31.5% (34) |
| New Balance | 32.6% (43) | 20% (27) | 40.7% (11) | 18.5% (5) | 30.5% (32) | 20.4% (22) |
| Brooks | 15.9% (21) | 22.2% (30) | 14.8% (4) | 11.1% (3) | 16.2% (17) | 25% (27) |
| Other | 54.5% (72) | 43% (58) | 51.9% (14) | 55.6% (15) | 55.2% (58) | 39.8% (43) |

* = Missing data: Participation in other weightbearing sport: n=2 control included (1 female, 1 male), n=2 surgery excluded prior to baseline (1 female, 1 male) missing. Participation in other non-weightbearing sport: n=1 control included (female), n=1 surgery included (male), n=1 surgery excluded prior to baseline (male) missing. Age started running: n=1 control included (male) missing. Average weekly running distance: n=1 surgery included (female) missing. Longest distance ever run: n=1 control excluded prior to baseline (male) missing. Upper-limb strength training: n=1 surgery included (male) missing. Lower-limb strength training: n=1 control included (male) missing. Core strength training: n=1 surgery included (male), n=1 surgery excluded prior to baseline (male) missing. Upper/lower-limb stretching: n=1 surgery included (male) missing. Shoe brand preference: n=1 control included (female) missing. Examples of weightbearing sports other than running: e.g. football, basketball, tennis. Examples of non-weightbearing sports: swimming, cycling.
