# Supplementary material for: Cohort profile: trajectory of knee health in runners with and without heightened osteoarthritis risk (TRAIL) in Australia—prospective cohort study
Source: BMJ Open. 2025 Sep 28;15(9):e101625. doi: 10.1136/bmjopen-2025-101625 (PMC12481359; doi:10.1136/bmjopen-2025-101625)
Supplement: Supplementary file 6 [file bmjopen-2025-101625.reviewer_comments.pdf]

PEER REVIEW HISTORY

BMJ Open publishes all reviews undertaken for accepted manuscripts. Reviewers are asked to complete a checklist review form and are provided with free text boxes to elaborate on their assessment. These free text comments are reproduced below.

ARTICLE DETAILS

Title (Provisional)

Cohort profile: Trajectory of knee health in runners with and without heightened osteoarthritis risk (TRAIL) in Australia – Prospective cohort study

Authors

De Oliveira Silva, Danilo; Mentiplay, Benjamin F; Girdwood, Michael; Haberfield, Melissa J; Bruder, Andrea; Culvenor, Adam; West, Tom J; Hill, Joshua Paul; Carey, David; Johnston, Richard TR; Crossley, Kay

---

VERSION 1 - REVIEW

**Reviewer**	**1**
**Name**	**OZTERMELI, Ahmet**
**Affiliation**	**Gebze Fatih State Hospital, Ortopedics and Traumatology**
**Date**	**08-May-2025**
**COI**	**None**

This is a well designed prospective cohort profile describing the TRAIL study, which investigates long-term knee health in runners with and without a history of knee surgery. The study addresses an important knowledge gap, with strong methodological foundations including clear inclusion criteria, balanced gender distribution, and high engagement through wearable technology.

Please consider discussing the potential differences in osteoarthritis risk profiles across the surgical subgroups (ACL reconstruction, meniscus, cartilage procedures...).

**Reviewer**	**2**
**Name**	**Capin, Jacob**
**Affiliation**	**Marquette University, Physical Therapy**
**Date**	**22-Jul-2025**

COI **Research funding (within the past 5 years): Marquette University Athletic and Human Performance Research Center Pilot Grant, NIH/OD DP5-OD031833 NIH Director’s Early Independence Research Award,**

Marquette University Institute for Women's Leadership Summer Interdisciplinary Grant, NIH/NIA F32-AG066274 Ruth L. Kirschstein National Research Service Award F32 Postdoctoral Fellowship, Academy of Orthopaedic Physical Therapy (AOPT) Career Development Award, Veterans Health Administration Eastern Colorado Geriatric Research, Education, and Clinical Center Advanced Geriatrics Fellowship

---

This study describes the cohort profile and baseline characteristics for an impressive, ongoing prospective cohort study following runners with and without a prior knee surgery in Australia. This study provides information on the 268 runners who enrolled in the study as well as the 214 who remained in the study and attended the baseline data collection. While the forthcoming longitudinal follow-up data and analyses will be significantly more intriguing and impactful, there is a need for transparently and comprehensively reporting baseline characteristics for prospective studies. The present manuscript does a good job of presenting these detailed baseline characteristics. Below are specific comments and questions to further strengthen this manuscript.

Specific comments:

ABSTRACT: please add more details to the abstract regarding: 1) the amount of time between enrollment and baseline testing; and 2) the planned longitudinal follow-up including biomechanical assessments.

INTRODUCTION: overall, this is clear and well-written. As noted above, please add to the abstract some more details included in paragraph 3 of the introduction.

Section 3.1 Recruitment Data: Why were 54 participants unable to attend the baseline session? Please provide the specific numbers for unable to contact and unable to come in for testing in Figure 1. Were there other reasons?

Recruitment & Enrollment: While there is adequate information regarding the targeted recruitment of female participants to balance the cohorts near the end of recruitment, more details are needed regarding the recruitment, selection, and enrollment of those with and without a knee surgery. How was 'matching' of the surgery group and control group achieved? Were the controls excluded prior to baseline testing in part to match the surgery group? It is otherwise ironic that the same number of surgery and control group participants were excluded prior to baseline.

Table 2: Please add BMI to table 2.

Table 3: Does the "meniscal" category under type of surgery include cartilage surgery too as stated in the text?

Section 3.2.3.b Knee-related medication use: Is this any medication use? Please clarify in the text.

Section 3.2.3.c Running behavior: Is running considered a sport in this context, or are these statistics referring only to other sports excluding running? Please also provide examples of weightbearing and nonweightbearing sports for clarity.

Table 5:

-It is interesting that some participants in the study are no longer meeting the 3x/week minimum frequency required for eligibility. Could the authors please discuss this further?

-For the frequency of running interval training, I recommend placing "Never" first so that the options are in order of least to greatest participation in interval training.

-For the additional training in the last month (e.g., upper-limb strength, lower-limb strength), does this include participants who performed these types of exercises only 1x per month or greater? Please clarify in the table.

Section 3.4 Baseline time-point: "The reference knee was predominantly the right knee across groups…" This seems overstated given that the values in the groups ranged form 50% to 58.2%. Please consider other words instead of "predominantly".

Strengths and Limitations: overall clear, comprehensive, and balanced

Thank you again for the opportunity to review this manuscript. I look forward to following the results of this exciting and need prospective cohort study!

VERSION 1 - AUTHOR RESPONSE

Reviewer #1:

1) Reviewer comment: *"This is a well-designed prospective cohort profile describing the TRAIL study, which investigates long-term knee health in runners with and without a history of knee surgery. The study addresses an important knowledge gap, with strong methodological foundations including clear inclusion criteria, balanced gender distribution, and high engagement through wearable technology."*

Author's reply: Thank you for your positive comments and constructive feedback to improve our manuscript.

2) Reviewer comment: *"Please consider discussing the potential differences in osteoarthritis risk profiles across the surgical subgroups (ACL reconstruction, meniscus, cartilage procedures...)."*

Author's reply: As the reviewer indicated, in the TRAIL cohort, the surgical group includes participants with differing surgical histories, categorised into three types/subgroups: ACL reconstruction, meniscus and/or cartilage procedures, and other. While these subgroups share a heightened risk of knee osteoarthritis compared to the general population, the magnitude and mechanisms of risk may differ. Although our cohort profile paper does not compare outcomes across these subgroups, the cohort's design will allow future analyses to explore these differences longitudinally.
We have addressed this comment in the discussion. Please, see below:

"Within the surgery group, risk profiles for knee osteoarthritis may differ across subgroups. While our cohort profile does not explore subgroup-specific outcomes, the TRAIL dataset is well-positioned to explore these differences longitudinally, which may inform tailored prevention and management strategies based on individual surgery profiles."

Reviewer #2:

1) Reviewer comment: "*This study describes the cohort profile and baseline characteristics for an impressive, ongoing prospective cohort study following runners with and without a prior knee surgery in Australia. This study provides information on the 268 runners who enrolled in the study as well as the 214 who remained in the study and attended the baseline data collection. While the forthcoming longitudinal follow-up data and analyses will be significantly more intriguing and impactful, there is a need for transparently and comprehensively reporting baseline characteristics for prospective studies. The present manuscript does a good job of presenting these detailed baseline characteristics. Below are specific comments and questions to further strengthen this manuscript.*"

Author's reply: Thank you for your positive comments and constructive feedback to improve our manuscript.

2) Reviewer comment: "*ABSTRACT: please add more details to the abstract regarding: 1) the amount of time between enrollment and baseline testing; and 2) the planned longitudinal follow-up including biomechanical assessments.*"

Author's reply: We have updated the abstract to:

(i) Specify the interval between enrolment and baseline testing: we now report that baseline testing occurred at a median of 33 weeks after enrolment.
(ii) Clarify the planned longitudinal follow-up: the biomechanical assessments are conducted at baseline only and will not be repeated at follow-up. Longitudinal follow-up consists of ongoing patient-reported outcomes and wearable-derived training-load monitoring, and at 4 and 10 years we will collect knee MRI and knee-health PROMs.

"Data were collected at study enrolment and at a face-to-face baseline testing session, which occurred a median of 33 weeks (interquartile range 18–86 weeks) after enrolment. Follow-up data collection is ongoing."

"*Future plans*: Participants will be followed for 10 years via ongoing patient-reported outcomes and continuous training-load monitoring from wearable devices. At 4 and 10-year follow-up, knee MRI and knee-health patient-reported outcomes will be collected to evaluate structural and symptomatic knee osteoarthritis progression. Data will inform guidelines for safe running practices and rehabilitation post-knee surgery."

3) Reviewer comment: *"INTRODUCTION: overall, this is clear and well-written. As noted above, please add to the abstract some more details included in paragraph 3 of the introduction."*

Author's reply: Thanks for your positive comments about our introduction. We have added the details to our abstract (response #2 above).

4) Reviewer comment: *"Section 3.1 Recruitment Data: Why were 54 participants unable to attend the baseline session? Please provide the specific numbers for unable to contact and unable to come in for testing in Figure 1. Were there other reasons?"*

Author's reply: Due to the six COVID-19 lockdowns in Melbourne across 2020 to 2022, there were significant interruptions to our face-to-face baseline data collection efforts, resulting in significant time delays between enrolment and baseline data collection for some participants. This might explain why we were unable to contact 37 participants to schedule or reschedule

their face-to-face baseline testing. Of the 17 participants we were able to contact, three reasons were cited for their inability to attend baseline lab testing: they could not commit to the required lab testing time commitment (n=3), they had moved interstate (n=4), or they explicitly stated they lost interest due to Covid-19 delays (n=10). We have updated Figure 1 to provide more detail for the reader (see below).

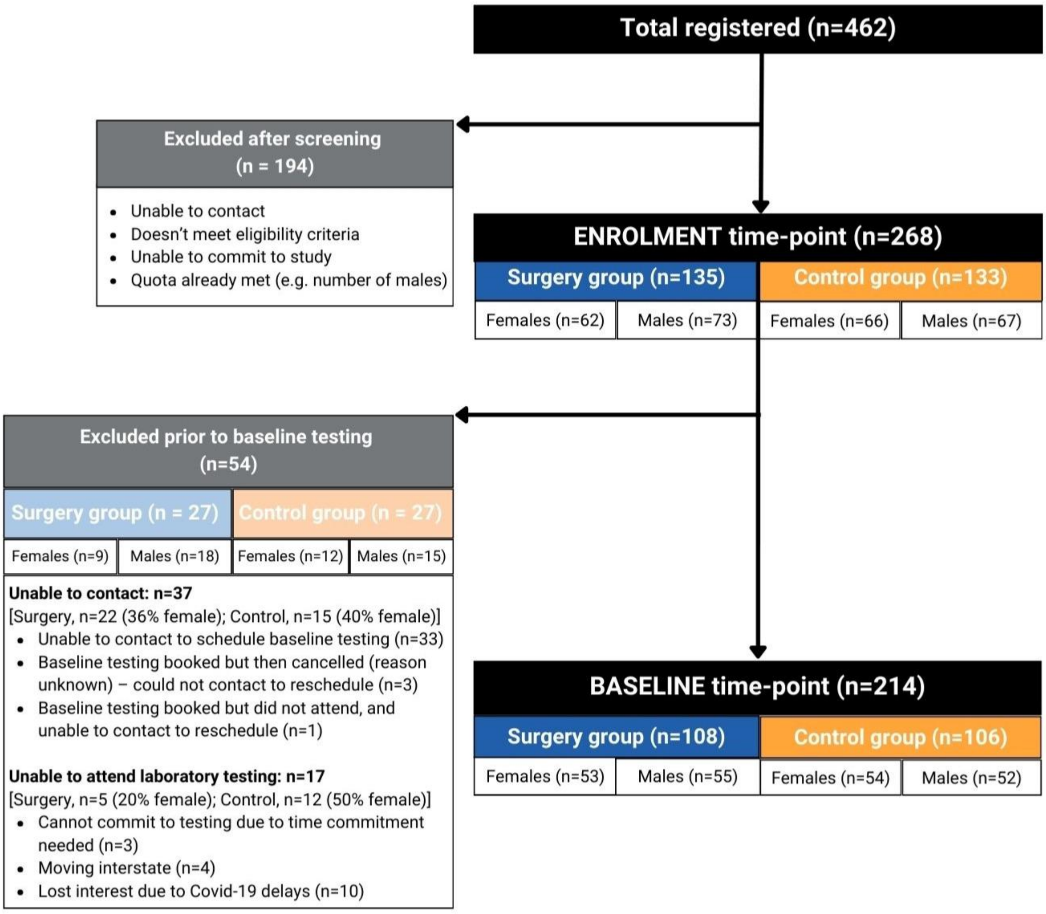

Figure 1: Flow chart describing the TRAIL participant's trajectory through the study.

5) Reviewer comment: *"Recruitment & Enrollment: While there is adequate information regarding the targeted recruitment of female participants to balance the cohorts near the end of recruitment, more details are needed regarding the recruitment, selection, and enrollment of those with and without a knee surgery. How was 'matching' of the surgery group and control group achieved? Were the controls excluded prior to baseline testing in part to match the surgery group? It is otherwise ironic that the same number of surgery and control group participants were excluded prior to baseline."*

Author's reply: We did not perform individual or frequency matching. Both groups were recruited through the same public channels and screened using identical criteria (other than surgical history). During screening, we targeted a 1:1 enrolment ratio between groups to support comparability, but no selective exclusions were undertaken after enrolment. During the screening process, we aimed to recruit an even number of participants with and without a history of knee surgery (see change in the manuscript below). Control participants were not excluded to match the surgery group. The equal number of pre-baseline withdrawals in each group (27 vs 27) was coincidental and due to participant-related logistics, mainly the six COVID-19 lockdowns in Melbourne and life change circumstances due to the pandemic, rather than investigator decisions. We have clarified these points in the manuscript sections noted below.

"We targeted a 1:1 enrolment ratio between the surgery and control groups to support balance; no formal matching was undertaken, and no post-enrolment exclusions were implemented to force balance."

6) Reviewer comment: "*Table 2: Please add BMI to table 2.*"

Author's reply: We have added BMI to Table 2.

7) Reviewer comment: "*Table 3: Does the "meniscal" category under type of surgery include cartilage surgery too as stated in the text?*"

Author's reply: Thanks for flagging this inconsistency. We have amended the wording of Table 3 to ensure consistency with the text.

8) Reviewer comment: "*Section 3.2.3.b Knee-related medication use: Is this any medication use? Please clarify in the text.*"

Author's reply: We have clarified this point in the text. Please, see below:

"3.2.3.*b. Medication use for knee pain*

Table 4 presents the self-reported use of any medication for knee pain reported by participants for the 3 months prior to enrolment time-point."

9) Reviewer comment: "*Section 3.2.3.c Running behavior: Is running considered a sport in this context, or are these statistics referring only to other sports excluding running? Please also provide examples of weight-bearing and non-weight-bearing sports for clarity.*"

Author's reply: The data regarding weightbearing and non-weightbearing sports do not include running, the details about running are included in the paragraphs 2 and 3 of this topic. We have reworded this topic to clarify the reviewer's comment. Please, see below:

"Table 5 presents characteristics related to participants' running behaviour, including running history, training practices, and participation in sports other than running at the enrolment time-point. Participants in the surgery group consistently reported higher participation in weight-bearing sports other than running (31.6%) compared to the control group (16.8%). Engagement in non-weight-bearing sports was also more frequent in the surgery group (50.4%) than in the control group (43.2%)."

We have added examples of weightbearing and non-weightbearing sports in the legend of the table, which can also be seen in the questionnaire provided in supplementary file 2. See below:

"Examples of weightbearing sports other than running: e.g. football, basketball, tennis. Examples of non-weightbearing sports: swimming, cycling."

10) Reviewer comment: "*Table 5:*
-It is interesting that some participants in the study are no longer meeting the 3x/week minimum frequency required for eligibility. Could the authors please discuss this further?"

Author's reply: This is a very insightful comment. At the time of recruitment, all participants met the eligibility criteria of running at least three times per week on average over the last 6 months. However, the frequency question in Table 5 refers to the past month only. It is possible that a participant met the eligibility criteria, but also reported lower running frequency in the previous month. This discrepancy likely reflects natural fluctuations in training behaviour due to factors such as injury, illness, life commitments, or seasonal variation, and is consistent with the longitudinal nature of the study where running exposure is expected to vary over time.

Importantly, these variations will be accounted for in longitudinal analyses using the objectively measured training load data from wearable devices.

We have added a discussion about this point. Please, see below:

"A large variability in the interval between enrolment and baseline laboratory testing (ranging from 22 to 92 weeks) could introduce some level of heterogeneity in baseline measures. Of note, although all participants met the ≥3 runs/week on average in the previous 6 months eligibility criterion at recruitment, some reported lower running frequency in the previous month at enrolment. This is likely due to natural fluctuations in running habits that may occur due to injury, illness, competing life demands, or seasonal variation. These variations reflect real-world running behaviour and will be accounted for in analyses using objectively measured running exposure from wearable devices."

11) Reviewer comment: "Table 5:

-For the frequency of running interval training, I recommend placing "Never" first so that the options are in order of least to greatest participation in interval training."

Author's reply: We amended Table 5 based on the reviewer's suggestion. Now 'never' is the first option displayed in the table.

12) Reviewer comment: *"Table 5:*

-For the additional training in the last month (e.g., upper-limb strength, lower-limb strength), does this include participants who performed these types of exercises only 1x per month or greater? Please clarify in the table."

Author's reply: Correct, this includes the participants who performed at least 1 session of additional training in the last month. We amended Table 5 based on the reviewer's suggestion. Please, see below:

Table 5: Additional training in the last month (At least 1 session).

13) Reviewer comment: *"Section 3.4 Baseline time-point: "The reference knee was predominantly the right knee across groups…" This seems overstated given that the values in the groups ranged form 50% to 58.2%. Please consider other words instead of "predominantly"."*

Author's reply: We have amended this sentence based on the reviewer's suggestion. Please, see below:

"The reference knee had an even distribution across the right and left sides."

14) Reviewer comment: *"Strengths and Limitations: overall clear, comprehensive, and balanced.*
Thank you again for the opportunity to review this manuscript. I look forward to following the results of this exciting and need prospective cohort study!"

Author's reply: Thanks for your comments and contributions to our paper.

VERSION 2 - REVIEW

**Reviewer**	**2**
**Name**	**Capin, Jacob**
**Affiliation**	**Marquette University, Physical Therapy**
**Date**	**26-Aug-2025**
**COI**	

Thank you for your clear and comprehensive revision in response to the initial review. I look forward to following the results of this important longitudinal study. Great work!
